# A224 COVID-19 INFECTION IN LIVER TRANSPLANT RECIPIENTS: CLINICAL FEATURES, HOSPITALIZATION, AND MORTALITY FROM A CANADIAN MULTICENTRE COHORT

**DOI:** 10.1093/jcag/gwab049.223

**Published:** 2022-02-21

**Authors:** M K Smith, J Chow, R Huang, M Omar, M Ebadi, P Wong, G Huard, E M Yoshida, D Peretz, M Brahmania, A J Montano-Loza, R Bhanji

**Affiliations:** 1 University of Alberta Faculty of Medicine & Dentistry, Edmonton, AB, Canada; 2 The University of British Columbia Faculty of Medicine, Vancouver, BC, Canada; 3 Gastroenterology, McGill University, Brossard, QC, Canada; 4 Liver diseases, Centre Hospitalier de l’Université de Montréal, Montreal, QC, Canada; 5 Division of Gastroenterology, University of British Columbia, Vancouver, BC, Canada; 6 University of Manitoba, Winnipeg, MB, Canada; 7 Western University, London, ON, Canada; 8 Division of Gastroenterology & Liver Unit, University of Alberta, Edmonton, AB, Canada

## Abstract

**Background:**

The COVID-19 pandemic has brought significant challenges to clinicians caring for liver transplant (LT) recipients. Researchers have sought to better understand the risk and clinical outcomes of LT recipients infected with COVID-19 globally, however, there is a paucity of data from within Canada.

**Aims:**

Our multi-center study aims to examine the characteristics and clinical outcomes of LT patients with COVID-19 in Canada.

**Methods:**

We identified a retrospective cohort of adult LT recipients with RT-PCR confirmed COVID-19 from 7 Canadian tertiary care centers between March 2020 and June 2021. Demographic and clinical data were compiled by clinicians within those centers. We identified liver enzyme profile at the time of COVID-19 infection, immunosuppression type and post-infection adjustments, rate of hospitalization, ICU admission, mechanical ventilation, and death.

**Results:**

A total of 49 patients with a history of LT and COVID-19 infection were identified. Twenty nine patients (59%) were male, the median time from LT was 66 months (1, 128) and the median age at COVID-19 infection was 59 years (52, 65). At COVID-19 diagnosis, the median ALT was 37 U/L (21, 41), AST U/L was 34 (20, 37), ALP U/L was 156 (88, 156), Total Bilirubin was 11 umol/L (7, 14), and INR was 1.1 (1.0, 1.1). The majority of patients (92%) were on tacrolimus monotherapy or a combination of tacrolimus and mycophenolate mofetil (MMF); median tacrolimus level at COVID-19 diagnosis was 5.3 ug/L (4.0, 8.1). Immunosuppression was modified in 8 (16%) patients post-infection; either the tacrolimus dose was reduced or MMF was held. One patient developed acute cellular rejection which recovered after re-initiation of the prior regimen. Eighteen patients (37%) required hospitalization, 6 (12%) were treated with dexamethasone, and 3 (6%) required ICU admission and mechanical ventilation. Four patients (8%) died due to complications of COVID-19. On univariate analysis, neither age, sex, co-morbidities nor duration post-transplant were associated with risk of hospitalization.

**Conclusions:**

In our national retrospective study, approximately 40% of patients required hospitalization with a mortality rate of < 10%. Previous studies have shown proximity to LT as an independent factor for mortality with COVID-19; the median time from LT for our patients was 5 years, which may explain the lower mortality rate. Of note, the median tacrolimus levels were much lower in comparison to the target of 8–10 ug/L used in the first year post-transplant. As the landscape of COVID-19 changes with vaccination, evolving treatments, and increasing rates of variant transmission, additional studies are required to continue identifying trends in clinical outcomes.

**Funding Agencies:**

None

